# Women with Down syndrome show more severe Alzheimer's disease neuropathology with regional differences

**DOI:** 10.1002/alz70855_107096

**Published:** 2025-12-24

**Authors:** Elizabeth J. Andrews, Phong T. Ngo, Jesse R. Pascual, Freddy Gonzalez, Michael J. Phelan, Sierra Wright, Jordan P. Harp, Frederick A Schmitt, Florence Lai, Patrick J. Lao, Adam Brickman, Julia K. Kofler, Milos D. Ikonomovic, Elizabeth Head, ABC‐DS Consortium

**Affiliations:** ^1^ University of California, Irvine, Irvine, CA, USA; ^2^ Alzheimer Biomarker Consortium‐Down Syndrome, Irvine, CA, USA; ^3^ University of Kentucky / Sanders‐Brown Center on Aging, Lexington, KY, USA; ^4^ University of Kentucky College of Medicine Department of Neurology, Lexington, KY, USA; ^5^ University of Kentucky College of Medicine Sanders‐Brown Center on Aging, Lexington, KY, USA; ^6^ Harvard/Massachusetts General Hospital, Boston, MA, USA; ^7^ Taub Institute for Research on Alzheimer's Disease and the Aging Brain, Vagelos College of Physicians and Surgeons, Columbia University, New York, NY, USA; ^8^ University of Pittsburgh, Pittsburgh, PA, USA; ^9^ University of Pittsburgh School of Medicine, Pittsburgh, PA, USA

## Abstract

**Background:**

Biological sex affects Alzheimer's disease (AD) progression in the neurotypical population, with women showing more rapid tau accumulation. People with Down syndrome (DS) develop AD neuropathology by the age of 40 years and represent the most common genetic cause of AD. Sex differences in AD neuropathology in Down syndrome (DS) have not been well characterized. We hypothesized that women with DS would show more severe AD neuropathology, indicated by elevated *p*‐tau in the occipital cortex (affected late in disease) relative to frontal cortex (affected earlier in AD), compared to men with DS.

**Method:**

Postmortem fixed brain tissue from 156 individuals (frontal/occipital regions) were examined by immunostaining serial sections for *p*‐tau (AT8) and Aβ (6E10). Groups were as follows: DS (*n* = 14/13, age 1‐39yr), DS with AD (DSAD) neuropathology (*n* = 18/19 age 42‐61yr), late‐onset AD (*n* = 15/16, age 72‐96yr) along with age‐matched controls (*n* = 50/47, age 2‐92yr).

**Result:**

The DSAD group had higher levels of *p*‐tau and Aβ compared to age‐matched controls in both regions. The DSAD group also had significantly elevated levels of *p*‐tau compared to the late‐onset AD group in the frontal (*p* <0.05) and the occipital cortices (*p* <0.001). Comparing neuropathology loads of *p*‐tau and Aβ in men and women with DSAD, we found non‐significant trends in elevated *p*‐tau in both the frontal and occipital cortices in women with DSAD compared to men with DSAD. After adjusting for the effect of age in Aβ and *p*‐tau, these markers were significantly positively correlated in the occipital cortex of women with DS (*p* <0.05, R^2^=0.3008), but not in men with DS (*p* >0.05, R^2^=0.1681).

**Conclusion:**

Together, these results suggest women with DSAD may have a more severe pathologic burden, specifically in the occipital cortex, an area typically affected later in AD pathogenesis, compared to men with DSAD. Future studies will be required to confirm this difference. Sex differences may also be region‐specific, and sex should be considered in design and interpretation of future clinical trials.

